# Dual roles of glycine betaine, dimethylglycine, and sarcosine as osmoprotectants and nutrient sources for *Vibrio natriegens*

**DOI:** 10.1128/aem.00619-25

**Published:** 2025-04-23

**Authors:** Heather E. Thomas, Katherine E. Boas Lichty, Gary P. Richards, E. Fidelma Boyd

**Affiliations:** 1 Department of Biological Sciences, University of Delaware 5972, Newark, Delaware, USA; 2 U.S. Department of Agriculture, Agricultural Research Service, Dover, Delaware, USA; Michigan State University, East Lansing, Michigan, USA

**Keywords:** compatible solutes, nutrients, marine bacteria, dimethyl glycine, sarcosine, catabolism clusters

## Abstract

Bacteria respond to osmotic stress by intracellularly accumulating low molecular weight compounds called compatible solutes, also known as osmolytes. Glycine betaine (*N*,*N*,*N*-trimethylglycine, GB) is a highly effective and widely available osmolyte used by bacteria, algae, and plants for abiotic stress protection. Here, we highlight the dual roles of GB, dimethyl glycine (DMG), and sarcosine for both osmoprotection and a less known role as sole carbon sources. First, we showed that the marine halophile *Vibrio natriegens* can grow in 1% to 7% NaCl and biosynthesize GB, ectoine, and glutamate and import GB, DMG, and sarcosine in response to osmotic stress. Betaine-carnitine-choline transporters (BCCTs) for the uptake of GB and DMG, but not sarcosine, were identified. Bioinformatics analyses uncovered homologs of GB, DMG, and sarcosine catabolism genes (*dgcAB_fixAB, gbcA, gbcB, purU, soxBDAG, glyA, glxA*) clustered in the *V. natriegens* genome, and these genes had a limited distribution among vibrios. We showed that *V. natriegens* ATCC 14048 grew on GB, DMG, and sarcosine as sole carbon sources, and gbcA and *dgcA* were required for growth. A contiguous catabolism cluster was present in a subset of *Vibrio fluvialis* strains, and we demonstrated the growth of *V. fluvialis* 2013V-1197 in DMG and sarcosine as sole carbon sources. Phylogenetic analysis revealed the catabolism cluster did not share a common ancestor among members of the family *Vibrionaceae*.

**IMPORTANCE:**

Compatible solutes are frequently the most concentrated organic components in marine organisms, allowing them to adapt to high saline environments as well as affording protection to other abiotic stresses. These organic compounds are significant energy stores that have been overlooked for their potential as abundant nutrient sources for bacteria. Our study characterized glycine betaine (GB), dimethyl glycine (DMG), and sarcosine catabolism genes and showed their efficient use as carbon and energy sources by marine halophilic vibrios.

## INTRODUCTION

Marine bacteria encounter a range of salt concentrations in their environment and must account for osmotic stress and the resulting changes in turgor pressure. To compensate for osmotic stress, bacteria have evolved different mechanisms in order to maintain cellular homeostasis (1–9). A common and widely used strategy is the intracellular accumulation of compatible solutes, also known as osmolytes (1, 2, 5, 7–10). Compatible solutes are small organic compounds that can be accumulated in molar concentrations without causing disruptions to essential cellular functions (1, 2, 5, 7–10). In hyper-osmotic environments, osmolytes are accumulated intracellularly by either uptake from the environment or biosynthesis. There are only a few examples of *de novo* osmolyte biosynthetic pathways present in bacteria, such as ectoine biosynthesis from its endogenous precursor L-aspartate. Most biosynthetic pathways require a precursor from the surrounding environment such as choline for glycine betaine (GB) biosynthesis (encoded by *betIBA*) (11–13). Transport of osmolytes into the cell is accomplished using specialized osmolyte transporters, members of the ATP-binding cassette (ABC) family that require ATP, secondary transporters of the betaine-carnitine-choline transporter (BCCT) family, the major facilitator superfamily, and the tripartite ATP-independent periplasmic family of transporters (14–24). Osmolyte transporters are predominantly expressed under high NaCl conditions and repressed when the osmotic stress is removed (17, 20–22, 24–27).

Previous studies characterized osmolyte uptake by *Vibrio cholerae*, *V. coralliilyticus*, *V. fluvialis, V. harveyi*, *V. parahaemolyticus,* and *V. vulnificus,* showing all species utilized GB as a highly effective osmoprotectant and biosynthesized GB from choline (21, 25, 28–32). Studies in *V. parahaemolyticus* showed optimal growth in up to 6% NaCl and the ability to biosynthesize ectoine, glutamate, and GB, and to uptake 15 different osmolytes under osmotic stress conditions, which included choline, GB, *N*,*N*-dimethylglycine (DMG), dimethylsulfoniopropionate (DMSP), and ectoine using high-affinity BCCT transporters for uptake (11, 21, 25, 29–31, 33).

**Fig 1:**
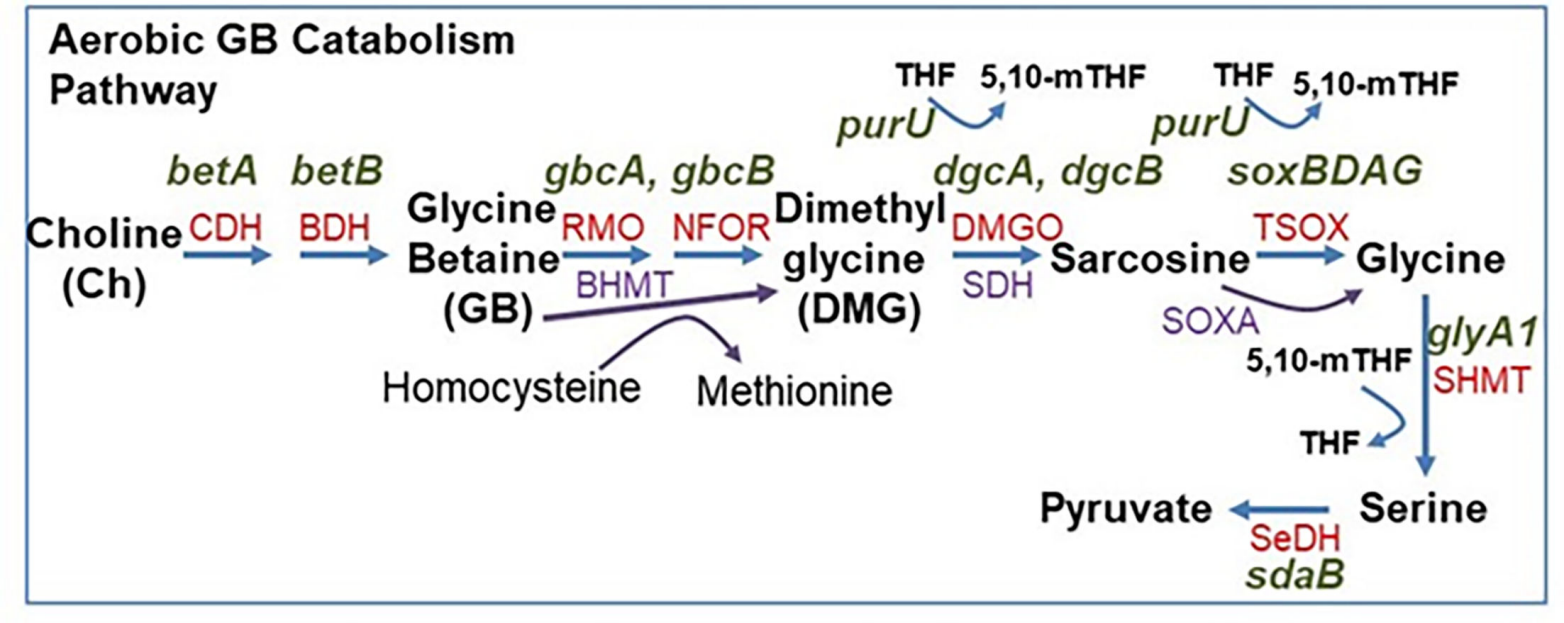
Glycine betaine aerobic catabolism pathway. Enzyme abbreviations in red and gene designations in black above are those present in *Pseudomonas aeruginosa*. Abbreviations: aerobic pathway in red, choline dehydrogenase (CDH, *betA*), betaine aldehyde dehydrogenase (BDH, *betB*), Rieske mono-oxygenase (RMO, *gbcA*), NADH:Flavin oxidoreductase (NFOR, *gbcB*), DMG oxidases (DMGO, *dgcAB*), heterotetrameric sarcosine oxidase (TSOX, *soxBDAG*). Glycine can be further broken down by serine hydroxymethyltransferase (SHMT, *glyA1*) and serine dehydratase (SeDH, *sdaB*) to pyruvate to enter central metabolism. Tetrahydrofolate (THF); 5,10-methylenetetrahydrofolate (5,10-mTHF). Enzyme abbreviations in purple represent alternative enzymes present in other bacteria such as *Sinorhizobium meliloti* that use BHMT to convert GB and homocysteine into DMG and methionine. Some *Bacillus* species use monomeric sarcosine oxidase (SOXA) to convert sarcosine to glycine.

Osmolytes are organic compounds and therefore have the potential to be used as carbon or nitrogen and energy sources (13, 30, 34). GB is an abundant osmolyte produced and utilized by bacteria, plants, and animals for stress protection (35–41). Levels of GB in marine environments range from picomolar in seawater and nanomolar in particulate matter to the mM range in phytoplankton and 100 mM range in corals (34–41). While the ability of bacteria to biosynthesize GB from choline is ubiquitous, their capacity to catabolize GB is much less common (13, 30, 34). A foundational study examining soil and activated sludge samples demonstrated that a large number of aerobic bacteria could utilize choline, GB, DMG, and sarcosine as sole carbon and nitrogen sources (42). That study showed that osmolyte degradation was found mainly in coryneforms and *Pseudomonas* genera (42). Aerobic catabolism of GB was subsequently demonstrated in *Corynebacterium*, *Arthrobacter*, *Sinorhizobium, Chromohalobacter, Halomonas*, and *Pseudomonas* species (17, 43–54). The aerobic catabolism pathway involved serial demethylation of GB to DMG to methylglycine (sarcosine) to glycine (Fig. 1) (17, 50, 52, 55–57). The first gene in the pathway is *gbcA,* which encodes a Rieske-family oxidase, while a related gene, *gbcB,* encodes a ferredoxin reductase recently described in *Chromohalobacter salexigens,* which together convert GB to DMG and formaldehyde (58, 59). In *Sinorhizobium meliloti,* this first step in GB catabolism involves the enzyme betaine homocysteine methyltransferase (BHMT), which converts GB and homocysteine to DMG and methionine (46, 47, 60). The next step is DMG conversion to sarcosine requiring dimethylglycine demethylase encoded by *dgcAB,* which is followed by sarcosine conversion to glycine by a tetrameric sarcosine oxidase (TSOX) encoded by *soxBDAG* in *Pseudomonas* species (Fig. 1). Glycine is further broken down by serine hydroxymethyltransferase (*glyA1*) and serine dehydratase (*sda*) to pyruvate to enter central metabolism (Fig. 1). In *Pseudomonas aeruginosa*, transcription of *gbcA, gbcB, dgcAB,* and *soxBDAG* requires the activators GbdR, an AraC transcription regulator, which is induced by GB and DMG, and SouR, which is induced by sarcosine (52, 56, 57, 61). Recent marine microbial community studies examining the fate of exogenous GB using metabolomics and transcriptomic analyses highlighted GB uptake and catabolism as an important source of carbon, nitrogen, and energy (62–65). Previous bioinformatic analyses identified homologs of several enzymes required for GB catabolism in *Vibrio natriegens*, a marine halophile first isolated in the salt marsh mud of Sapelo Island, Georgia, USA, and a member of the ubiquitous Harveyi clade (30, 66).

In this study, we describe the osmotic stress response of *V. natriegens* to determine its NaCl tolerance and the mechanisms it uses to adapt to salinity. Members of the family *Vibrionaceae* have not been shown previously to utilize GB, DMG, or sarcosine as carbon and energy sources, which was also examined here. First, we determined the range of salinities in which *V. natriegens* could grow and whether NaCl played a role in temperature tolerance. Next, we examined the range of osmolytes that *V. natriegens* could utilize in response to osmotic stress. Using a bioinformatic approach, we examined the genome of *V. natriegens* ATCC 14048 for homologs of previously identified osmotic stress systems. Proton nuclear magnetic resonance (^1^H-NMR) spectroscopy was used to determine the compatible solute biosynthesized by this species. Osmolyte uptake assays of stressed cells were used to examine osmolyte transport and the role individual BCCT transporters played. Genome context and gene neighborhood analyses identified a BCCT transporter clustered with genes showing homology to enzymes involved in the conversion of GB to pyruvate. The ability to consume GB, DMG, and sarcosine as carbon and energy sources was examined in *V. natriegens* and *V. fluvialis*. The distribution of the catabolism cluster was examined among *Vibrionaceae*.

## RESULTS

### *Vibrio natriegens* response to salinity and temperature

**Fig 2:**
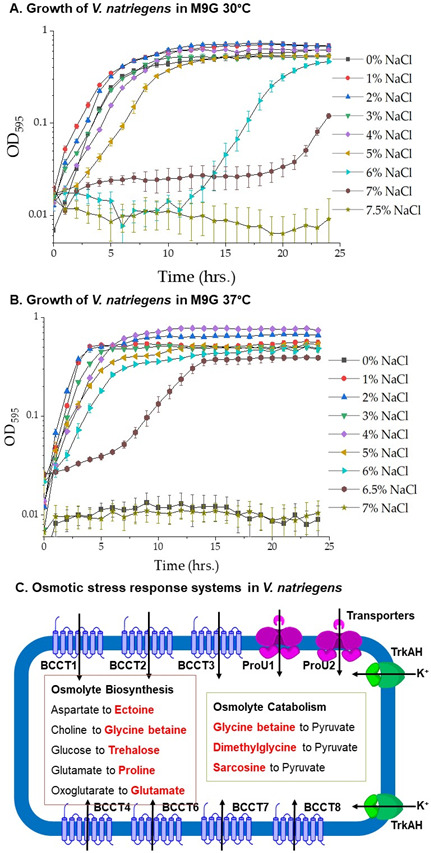
*Vibrio natriegens* ATCC 14048 NaCl tolerance range. Growth curves of *V. natriegens* ATCC 14048 in minimal media (**M9**) with glucose (M9G) with a final concentration of 1% to 7.5% NaCl. Growth was measured every hour for 24 h at (**A)** 30°C, (**B)** 37°C, and (**C)** osmotic stress response systems identified in *V. natriegens* ATCC 14048. Depicted are two TrkAH transporters for the uptake of potassium, a short-term response to osmotic stress, seven BCCT transporters identified, and two ABC-type transporters, ProU1 and ProU2, encoded by *proVWX*. Also shown are osmolyte biosynthesis pathways and osmolyte catabolic pathways identified. Arrows show the direction of substrate transport.

*Vibrio natriegens* ATCC 14048 was grown in lysogeny broth (LB) 3% NaCl (wt/vol) at 37°C for 24 h. A short lag phase was observed with the cultures growing to an optical density (OD_595_) of ~1.55 in less than 2.5 h, with a growth rate of 1.9 confirming previous studies of its fast growth rate (data not shown) (66–68). To determine the NaCl tolerance range of *V. natriegens*, we examined growth in M9 minimal media supplemented with 20 mM glucose (M9G) and 0% NaCl to 7.5% NaCl in the absence of external osmolytes at 30°C. *Vibrio natriegens* grew optimally in 0% to 5% NaCl; whereas at 6% and 6.5% NaCl, they grew after extended lag phases, while at 7% NaCl, no growth was observed (Fig. 2A). At 37°C, *V. natriegens* grew optimally between 1% and 6% NaCl; at 7% NaCl, it had an extended lag phase, and at 7.5% NaCl, there was no growth (Fig. 2B). In addition, *V. natriegens* did not grow in the absence of NaCl at 37°C. These data indicate that *V. natriegens* has a higher NaCl tolerance range at 37°C but also has a requirement for NaCl to grow at this temperature, indicating that NaCl plays a role in thermal tolerance.

### Osmotic stress response systems in *V. natriegens*

Bioinformatics analysis was performed comparing osmotic stress systems present in *V. parahaemolyticus* RIMD 2210633 to the *V. natriegens* ATCC 14048 genome RefSeq GCF_001456255.1. BLAST analysis uncovered two putative osmolyte biosynthesis operons for ectoine (encoded by *ectABC_aspK*) and glycine betaine (GB) (encoded by *betIBA*) as well as nine putative osmolyte transporters (Fig. 2C). Chromosome I (NZ_CP009977.1) contained two BCCTs, BCCT1 and BCCT3 and a ProU transporter (encoded by *proVWX*), which was adjacent, but divergently transcribed from *ectABC_aspK*. Chromosome II (NZ_CP009978.1) contained five BCCTs, BCCT2 (PN96_RS23610), BCCT4 (PN96_RS17645), BCCT6 (PN96_RS15305), BCCT7 (PN96_RS21680), and BCCT8 (PN96_RS22655), and a ProU transporter (*proXWV*, PN96_RS19360-PN96_RS19370) in an operon *betIBA_proXWV*. The numbering of BCCT1 to BCCT4 is based on their homology to the BCCTs from *V. parahaemolyticus* RIMD 2210633. BCCT5 was identified previously in *Vibrio vulnificus* and is absent from *V. natriegens* (29). Therefore, when numbering the additional BCCTs present in *V. natriegens*, BCCT5 was skipped. Overall, the *V. natriegens* ATCC 14048 genome contains seven BCCTs, whereas strains CL-2, TC2-11, and SY.PD55 contained eight BCCTs, four more than are present in *V. parahaemolyticus*.

### Ectoine, glutamate, and glycine betaine are biosynthesized by *V. natriegens* for osmotic stress protection

**Fig 3:**
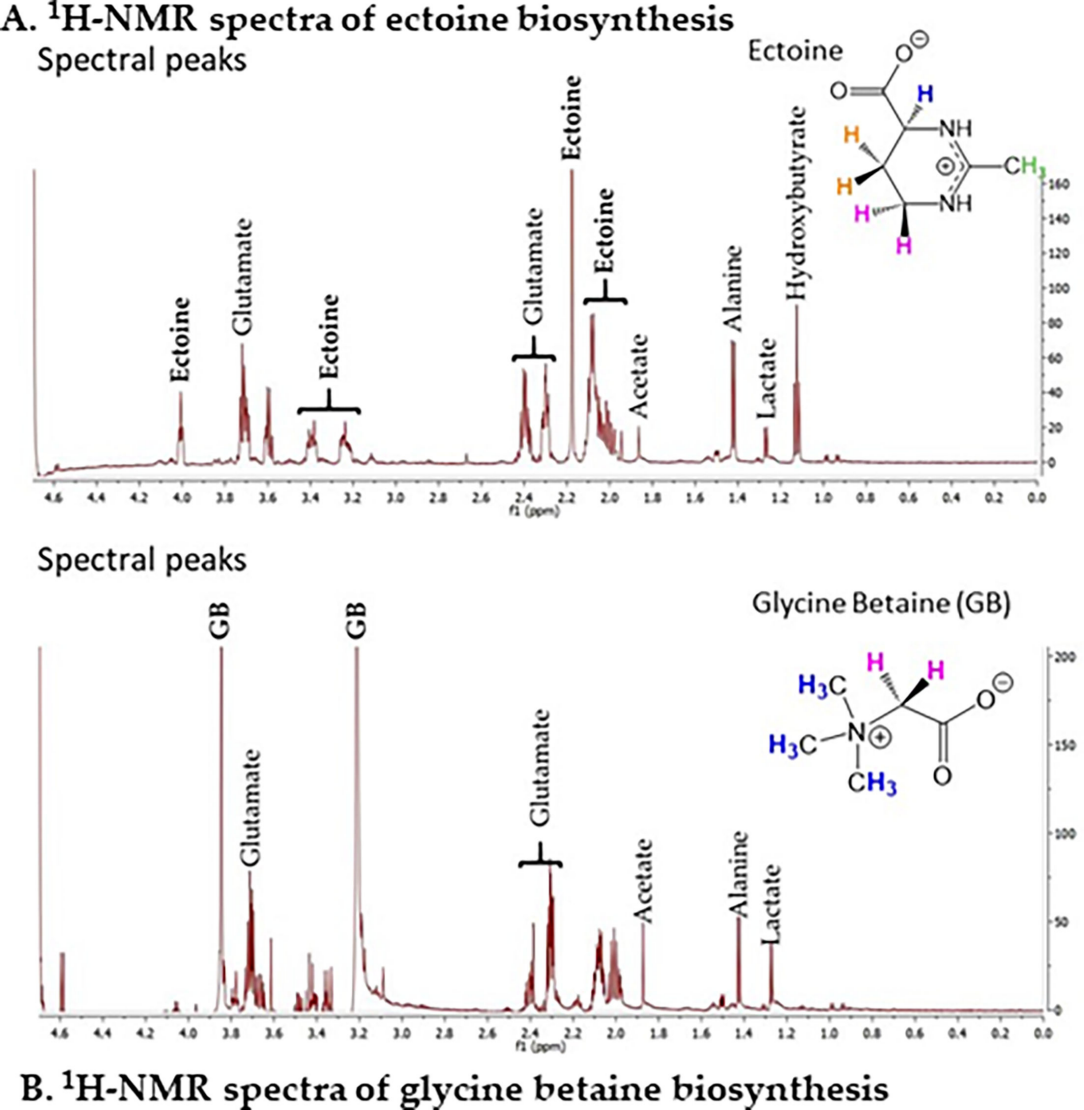
Biosynthesis of compatible solutes in *Vibrio natriegens* ATCC 14048. (A) ^1^H-NMR spectra of *V. natriegens* ATCC 14048 cellular extract grown in minimal media (M9G) 5% NaCl and (**B)** M9G 5% NaCl with the addition of 1 mM choline. The spectral peaks corresponding to glutamate, ectoine, and GB are labeled. The protons' spectral peaks corresponding to (**A)** glutamate (peaks at 3.7, 2.4, 2.3 ppm) and ectoine (4.0, 3.4, 3.25 ppm, 2.1–2.2 ppm singlet, 2.0–2.1 ppm multiplet) and (**B)** GB (3.85, 3.2 ppm) and glutamate (peaks at 3.7, 2.4, 2.3 ppm) are labeled, as illustrated by their chemical shift values expressed in ppm on the x-axis.

The putative ectoine biosynthesis operon *ectABC*_*aspK* should allow for the biosynthesis of ectoine *de novo* from endogenous aspartic acid (Fig. 2C). To examine this, ^1^H-NMR spectroscopy was performed on ethanol extracts of *V. natriegens* grown in M9G 5% NaCl. The spectral peaks corresponding to the various hydrogen atoms in the ectoine molecule are labelled in Fig. 3A. This analysis also identified peaks corresponding to the osmolyte glutamate, indicating it is also produced in response to osmotic stress (Fig. 3A). As a negative control, cells were also grown in M9G 1% NaCl, and extracts from these unstressed cells lacked ectoine and glutamate proton peaks (Fig. S1). Next, *V. natriegens* was examined for its ability to biosynthesize GB from its exogenously supplied precursor choline. ^1^H-NMR spectra of ethanol extracts of *V. natriegens* grown in M9G 5% NaCl supplemented with 1 mM choline showed signal peaks corresponding to GB (Fig. 3B). Ectoine peaks were absent in these spectra, demonstrating ectoine is not biosynthesized in the presence of choline (Fig. 3B). Overall, the data showed that *V. natriegens* was able to biosynthesize both glutamate and ectoine *de novo* and GB in the presence of choline in response to osmotic stress.

### *Vibrio natriegens* can uptake and utilize a range of osmolytes for osmoprotection

**Fig 4:**
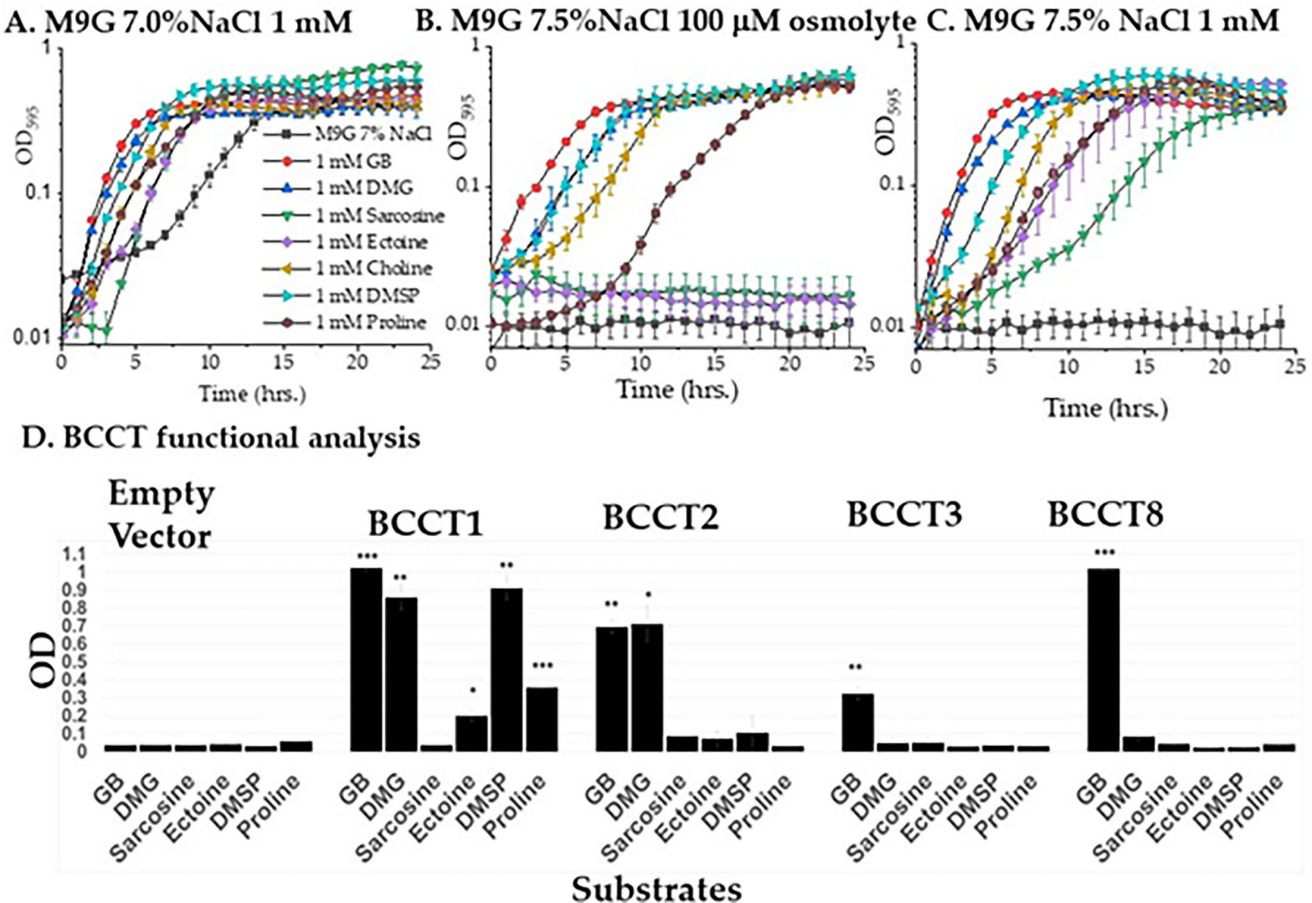
Import of compatible solutes for osmotic protection in *Vibrio natriegens* ATCC 14048. (A) *V. natriegens* ATCC 14048 growth in M9G 7% NaCl with 1 mM GB, DMG, sarcosine, ectoine, choline, DMSP, or L-proline. (**B)** *V. natriegens* growth in M9G 7.5% NaCl in 100 µM GB, DMG, sarcosine, ectoine, choline, DMSP, or L-proline and (**C)** 1 mM GB, DMG, sarcosine, ectoine, choline, DMSP, or L-proline. OD was measured for 24 h at 37°C. (D**)** Functional complementation of *Escherichia coli* MKH13 harboring *bccT1*, *bccT2*, *bccT3*, and *bccT8* grown in M9G–4% NaCl medium in the absence or presence of exogenously supplied compatible solutes GB, DMG, sarcosine, ectoine, DMSP, or L-proline. The error bars represent means ± standard errors for two biological replicates. Statistical analysis was performed by comparing the *bccT*-complemented *E. coli* strain to the corresponding empty vector supplemented with the same compatible solute. ****, *P* ≤ 0.001.

To determine the range of osmolytes taken up and utilized by *V. natriegens* for osmoprotection, cells were grown in M9G 7% NaCl supplemented with 1 mM of choline, GB, DMG, sarcosine, DMSP, ectoine, or L-proline. In the absence of osmolytes, there was a 6 h lag phase, which was significantly reduced in the presence of 1 mM of all solutes examined, with GB, DMG, and DMSP showing the greatest lag phase reduction (Fig. 4A). Next, these osmolytes were examined to determine whether they restored growth in M9G 7.5% NaCl, a condition that inhibits growth. M9G 7.5% NaCl was supplemented with 100 µM of each osmolyte, and GB, DMG, DMSP, choline, and L-proline rescued growth. The length of the lag phase for each substrate suggests there may be differences in transporter affinities. For example, GB has the shortest lag phase, indicating it is taken up efficiently, whereas L-proline has a 5 h lag phase, suggesting a less efficient transporter. Ectoine and sarcosine did not rescue growth at this concentration (Fig. 4B). When the concentration was increased to 1 mM, all seven osmolytes rescued growth, with sarcosine having the longest lag phase, but rescues to wild-type levels (Fig. 4C). This suggests that sarcosine does not have an efficient transporter for uptake in high NaCl. Overall, these data indicate that *V. natriegens* was able to uptake and utilize osmolytes as osmoprotectants and that choline, GB, DMG, and DMSP are most effective based on their shorter lag phases and ability to rescue growth at low concentrations.

### BCCT1, BCCT2, BCCT3, and BCCT8 can transport osmolytes into the cell

Seven BCCTs were present in the genome of *V. natriegens,* and to determine the functionality of each, we cloned each *bccT* gene into *Escherichia coli* MKH13. The MKH13 strain of *E. coli* is a *putP, proP, proU,* and *betT-betIBA* deletion mutant, which prevents it from transporting osmolytes and converting choline into GB, which prevents growth in 4% NaCl (4). Functional complementation of *E. coli* MKH13 was performed by first cloning each *bccT* gene into the expression plasmid pBAD and then transforming each into *E. coli* MKH13. An empty vector plasmid was also cloned into *E. coli* MKH13 as a negative control and showed no growth in 4% NaCl in the presence of all osmolytes tested (Fig. 4D). *E. coli* pVn*bccT1*, pVn*bccT2*, pVn*bccT3*, and pVn*bccT8* were able to grow in the presence of GB, indicating these BCCTs transported GB into the cell for osmoprotection (Fig. 4D). *E. coli* pVn*bccT1* was able to uptake GB, DMG, DMSP, ectoine, and L-proline (Fig. 4D), and *E. coli* pVn*bccT2* transported GB, DMG, and DMSP (Fig. 4D). None of the transporters examined were able to uptake sarcosine, indicating a different transporter system is responsible (Fig. 4D).

Additionally, *E. coli* pVn*bccT4*, pVn*bccT6*, and pVn*bccT7* were unable to uptake any of the osmolytes tested (data not shown). Previous work by Gregory et al. characterized the GB binding pocket in BCCT1 from *V. parahaemolyticus* (31). BCCTs all contain 12 transmembrane (TM) domains, TM1 to TM12, and in *V. parahaemolyticus* BCCT1, the GB binding pocket residues were identified in TM4 and TM8. The specific amino acids identified as crucial for GB binding were Trp203, Trp208, and Tyr211 in TM4, and Trp380, Trp381, and Trp384 in TM8 (31). Our bioinformatics analysis of BCCT1, BCCT2, BCCT3, and BCCT8 from *V. natriegens* identified each of these residues in the corresponding TM4 and TM8 regions (Fig. S2). However, analysis of BCCT4, BCCT6, and BCCT7 showed a change in at least one of these essential residues (Fig. S2). For example, BCCT4, BCCT6, and BCCT7 had a change in the Tyr residue of the GB binding pocket in TM4, and BCCT4 and BCCT7 had a change in the second and third Trp residue of TM8 (Fig. S2). These changes to the GB binding pocket may be the cause of the inability of BCCT4, BCCT6, and BCCT7 to uptake GB. Since their expression and stability were not tested, it is not possible to rule out their transport.

### *Vibrio natriegens* ATCC 14048 can utilize GB, DMG, and sarcosine as sole carbon sources

**Fig 5:**
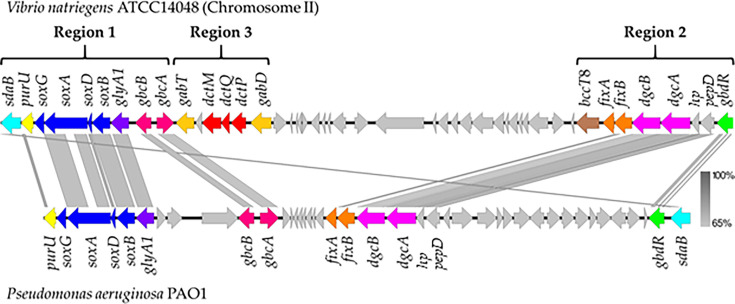
Genome comparison. Comparative analysis between *Vibrio natriegens* and *Pseudomonas aeruginosa* characterized GB catabolism genes. Arrows indicate open reading frames (ORFs) and the direction of transcription. Similarly colored arrows indicate genes with similar functions. Gray vertical bars indicate homology between genes.

In *V. natriegens* ATCC 14048, the genomic context of *bcct8 (PN96_RS22655*) presence in chromosome 2 was examined. The *bcct8* gene formed a cluster, with the operon *dgcAB_fixAB (PN96_RS22675* to *PN96_RS22655),* four genes previously identified in *Pseudomonas aeruginosa* PAO1 (Table S1) (Fig. 5) (50). The *fixAB* genes were predicted to encode heterodimeric electron transfer flavoprotein alpha and beta subunits. The *dgcA* gene (*PN96_RS22675*) encodes a protein with a NADH:flavin oxidoreductase domain, while *dgcB* (*PN96_RS22670*) encodes a protein with a Fe-S cluster containing an oxidoreductase domain and a domain of unknown function DUF3483 (Table S1). The *dgcAB* genes encode the alpha and beta subunits of a DMG dehydrogenase involved in the demethylation of DMG to sarcosine. Two additional genes preceded the *dgcAB_fixAB* cluster, encoding a hypothetical protein and a predicted dipeptidase (*pepD*). These two genes are also present at the same location in *P. aeruginosa* (Fig. 5) (50). Approximately 28 kb downstream of *pepD_hp_dgcAB_fixAB* was a second gene cluster (*PN96_RS22500* to *PN96_RS22455*) that contained genes encoding proteins required for catabolism of GB to DMG (*gbcA, gbcB*), sarcosine to pyruvate (*soxBDAG, glyA1, sdaB*), and the *purU* gene (Fig. 5). PurU is known to convert methyltetrahydrofolate (mTHF), a byproduct of the *dgcAB* and *soxBDAG* enzymatic reactions, into tetrahydrofolate (THF) (Table S1) (48, 69). The region that separated the two GB catabolism clusters in *V. natriegens* contained gene homologs involved in the α-aminobutyrate (GABA) shunt pathway (*gabT, gabD*), amongst others (Fig. 5).

**Fig 6:**
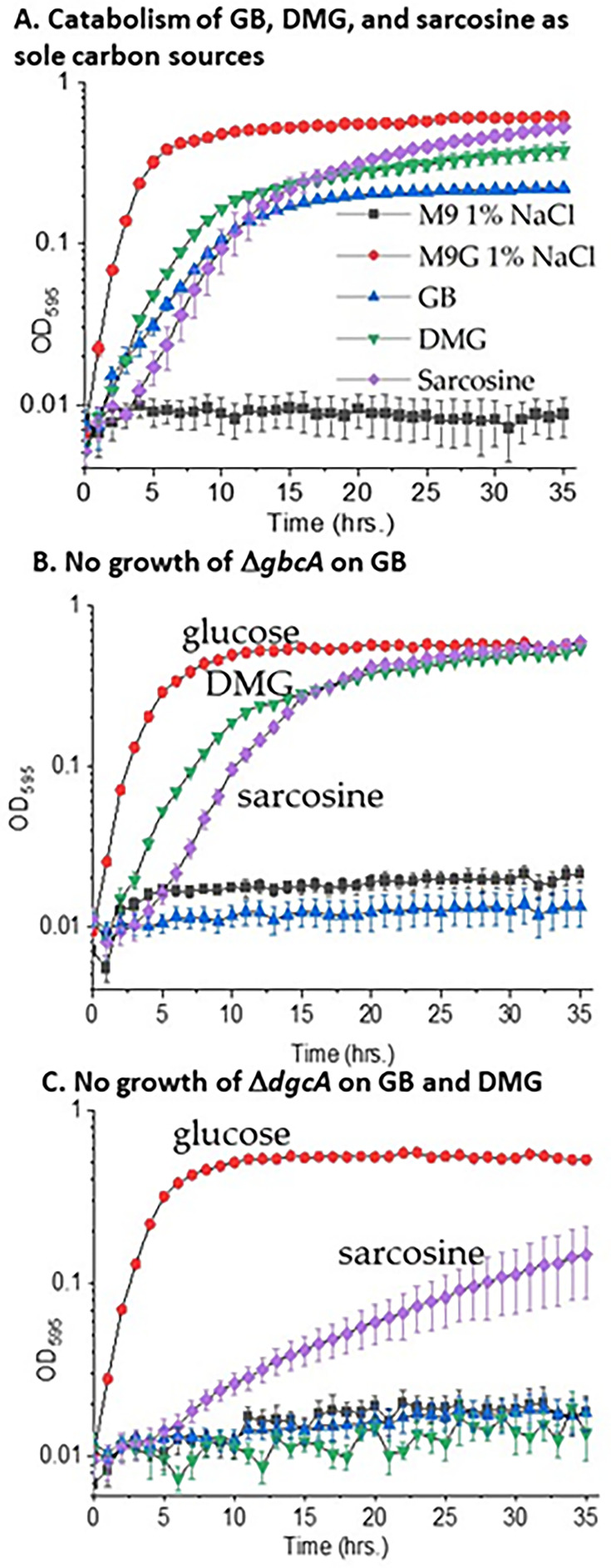
Utilization of GB, DMG, and sarcosine as sole carbon sources by *Vibrio natriegens***.** (A) Growth curves of *V. natriegens* ATCC 14048 in minimal media (**M9**) 1% NaCl at 30°C containing 20 mM glucose (M9G) or 20 mM GB, DMG, sarcosine, or no carbon source (**M9**) after 35 h. (**B)** Growth curves of the *V. natriegens gbcA* deletion mutant in M9 1% NaCl at 30°C on glucose (M9G), GB, DMG, and sarcosine. (**C)** Growth curves of the *V. natriegens dgcA* deletion mutant in M9 1% NaCl at 30°C in glucose (M9G), GB, DMG, and sarcosine.

To determine whether *V. natriegens* can grow on GB as a sole carbon source, growth was examined at 37°C in M9 supplemented with 3% NaCl or 1% NaCl with 20 mM GB as a sole carbon source. Under these conditions, no growth was observed (data not shown). Next, we examined growth at 30°C in M9 1% NaCl supplemented with 20 mM GB as sole carbon sources. Growth pattern analysis showed *V. natriegens* grew in GB with an approximately 1 h lag phase compared to M9G and a final OD of 0.22 (Fig. 6A). Next, we examined growth in DMG or sarcosine as sole carbon sources. In DMG, there was an approximately 1 h lag phase with a final OD of 0.38; whereas, on sarcosine, there was a 4 h lag phase with a final OD of 0.53. *V. natriegens’* growth on sarcosine compared favorably with growth on glucose, which had a final OD of 0.61, indicating a similar biomass is produced on each substrate (Fig. 6A). Examination for growth on M9 1% NaCl supplemented with choline as a sole carbon source showed no growth (data not shown). To demonstrate the requirement for *gbcA* in the initial demethylation of GB into DMG in *V. natriegens*, we generated a *gbcA* non-polar in-frame deletion mutant by splice overlapping extension (SOE) PCR and allelic exchange. The *V. natriegens* Δ*gbcA* mutant was grown at 30°C in M9 1% NaCl supplemented with 20 mM glucose, GB, DMG, or sarcosine. Growth of the mutant in M9G was similar to wild type indicating the mutant had no overall growth defect (Fig. 6B). The Δ*gbcA* mutant grew in media supplemented with DMG or sarcosine with an OD similar to growth on M9G but was unable to use GB as a sole carbon source. These data confirmed *gbcA* is required for GB catabolism but was not required for the breakdown of DMG and sarcosine, and the mutant does not exhibit any overall growth defect (Fig. 6B). This demonstrates that in *V. natriegens*, *gbcA* is only involved in the initial breakdown of GB into DMG, confirming the first step of our putative catabolism pathway in *V. natriegens*.

The second step of GB catabolism, DMG degradation to sarcosine, was accomplished by the *dgcAB* genes in *P. aeruginosa*. In *V. natriegens*, a Δ*dgcA* mutant was constructed and examined for growth on M9G, M9 GB, M9 DMG, or M9 sarcosine. The Δ*dgcA* mutant grew on M9G similar to the wild type, showing no overall growth defect by the mutant (Fig. 6C). The mutant failed to grow in M9 GB or M9 DMG but grew on M9 sarcosine with a lower OD compared to M9G. This established that in *V. natriegens, dgcA* was involved in the breakdown of DMG to sarcosine, confirming the second step in the GB catabolism pathway.

### *Vibrio fluvialis* 2013V-1197 can utilize DMG and sarcosine as sole carbon sources

**Fig 7:**
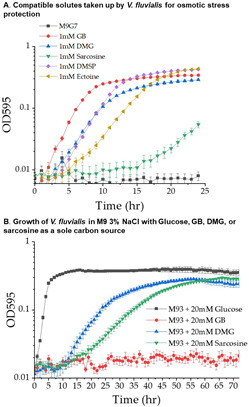
Compatible solutes as osmoprotectants and carbon sources in *V. fluvialis*. (A) Import of compatible solutes for osmotic stress protection. *V. fluvialis* 2013V-1197 grown in M9G 7.0% NaCl supplemented with 1 mM GB, DMG, sarcosine, DMSP, or ectoine. Growth was measured for 24 h at 37°C. (**B)** Catabolism of compatible solutes. Growth curves of *V. fluvialis* 2013V-1197 in M9 3% NaCl at 37°C on glucose (M9G), GB, DMG, and sarcosine after 70 h.

The catabolic genes were present as a single contiguous cluster among a subset of strains (39 strains) of *V. fluvialis,* an emerging seafood-borne pathogen that causes sporadic outbreaks of gastroenteritis worldwide (70). *Vibrio fluvialis* had a similar repertoire of osmotic stress response systems as other vibrios, containing the gene clusters for the biosynthesis of ectoine and GB as well as four BCCT transporters, BCCT1, BCCT2, BCCT3, and BCCT9 (no BCCT8 homolog was present) and two ProU transporters (Fig. S3A). *Vibrio fluvialis* 2013V-1197 showed growth in M9G 1% to 6% NaCl, with growth prohibited in M9G 7% NaCl at 37°C (Fig. S3B). This species could uptake GB, DMG, DMSP, and ectoine to rescue growth in M9G 7% NaCl (Fig. 7A). Sarcosine did not rescue growth under the conditions examined (Fig. 7A).

The ability of *V. fluvialis* 2013V-1197 to grow in M9 supplemented with GB, DMG, or sarcosine as sole carbon sources was examined in M9 1% NaCl at 30°C and 37°C. No growth was observed under these conditions (data not shown). Next, we examined growth in M9 3% NaCl at 30°C and 37°C supplemented with choline, GB, DMG, or sarcosine (Fig. S4A and B). No growth occurred in choline or GB as a sole carbon source after 72 h at 30°C or 37°C. At 30°C, growth occurred in DMG or sarcosine after 48 h, whereas at 37°C, growth occurred in DMG after 24 h (Fig. S4A and B). Next, growth curve analyses were performed in M9 3% NaCl supplement with GB, DMG, or sarcosine. In M9 3% NaCl supplemented with DMG, the growth curves showed a lag phase of 12 h with a final OD of 0.27 (Fig. 7B). In M9 3% NaCl supplemented with sarcosine, there was a lag phase of 14 h and a final OD of 0.27 after 60 h (Fig. 7B). Overall, these data show *V. fluvialis* 2013V-1197 cannot use GB as a sole carbon source but can consume either DMG or sarcosine as a sole carbon source.

### Phylogenetic analysis of DgcA and genome neighborhood analysis among marine bacteria

**Fig 8:**
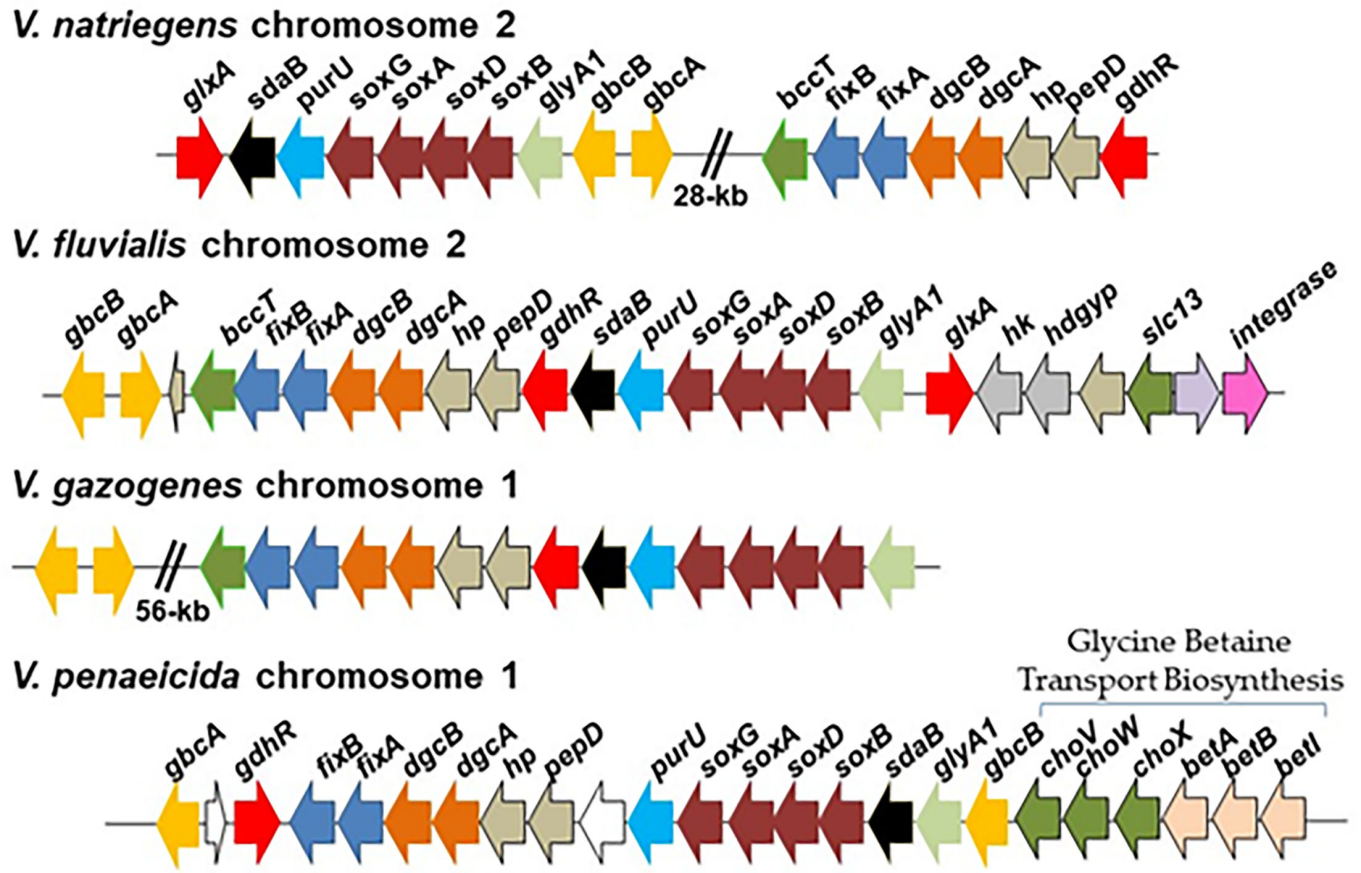
Schematic of the gene order of GB, DMG, and sarcosine transporter, catabolism, and regulatory genes in *Vibrio* species. Arrows indicate ORFs and direction of transcription. Similarly colored arrows indicate genes sharing a similar function, with the exception of white arrows that represent genes of unknown function in most cases. In *V. penaeicida*, the GB biosynthesis genes (*betIBA*) and transporter (*choXWV*) genes are directly upstream of GB catabolism genes.

The GbcA and DgcA proteins among *V. natriegens* strains shared >99% identity and were present in all strains in chromosome 2 at the same location, but were present in two chromosomal regions (Fig. 8). Whereas among other *Vibrio* species, the catabolism genes were present as a contiguous cluster (Fig. 8). In *V. fluvialis* 2013V-1197, the r GB catabolism genes were present in chromosome 2, and in *V. gazogenes*, *V. mangrovi, V. ruber,* and *V. spartinae* strains, the region was present in chromosome 1 (Fig. 8). In *V. fluvialis* 2013V-1197, the region was flanked on either side by integrases inserted close to core genes, a D-alanine–D-alanine ligase on one side and H-NS on the other. An IS4 element was also noted near the GB catabolism region in *V. ruber* and in *V. gazogenes* and *V. spartinae*. The region is flanked on one side by Rhs proteins and a few type VI secretion system genes and inserted adjacent to an asparagine-tRNA ligase in these species. This is also the insertion site for the region in *V. mangrovi*, but no integrases, IS elements, or Rhs proteins were present. In all *V. penaeicida* strains, the GB biosynthesis genes (*betIBA*) and transporter genes (*proVWX/choVWX*) were directly upstream of the GB catabolic genes also in chromosome 1 (Fig. 8). In *V. ostrea* OG9-811, the *glyA1_soxBDAG_purU_sdaB* genes were present in chromosome 1, and *gbcA, gbcB, pepD*_*hp_dgcAB_fixAB* genes were present in chromosome 2. These data suggest the independent acquisition of the region among species.

**Fig 9:**
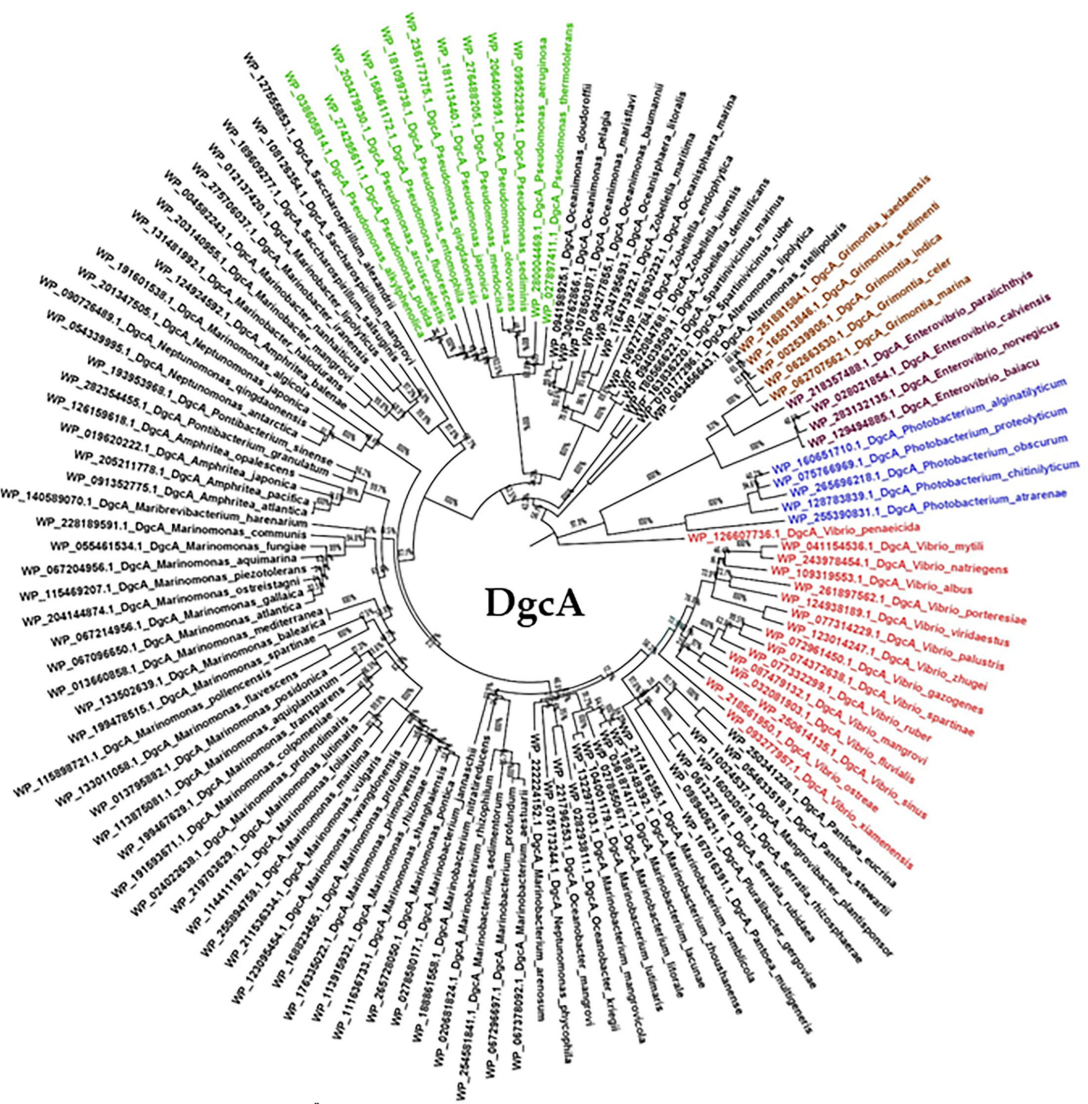
The evolutionary history of DgcA among *Vibrionaceae* using the neighbor-joining method with distances computed using the JTT matrix-based method and is in the units of the number of amino acid substitutions per site. The percentage of replicate trees in which the associated taxa clustered together in the bootstrap test (1,000 replicates) are shown next to the branches. This analysis involved 126 amino acid sequences, and ambiguous positions were removed using the pairwise deletion option. Shown in red are *Vibrio* species, purple *Enterovibrio*, brown *Grimontia*, blue *Photobacterium*, and green *Pseudomonas* species (only a subset of species from this genus is shown).

To further uncover the evolutionary history of GB, DMG, and sarcosine catabolism among *Vibrionaceae*, a phylogeny based on the DgcA protein was constructed (Fig. 9). The phylogeny placed DgcA from *Vibrionaceae* species within two highly divergent branches, with one branch comprised of *Vibrio* species sharing a most recent common ancestor with several *Pantoea* and *Serratia* species (Fig. 9). In these two genera, the GB catabolism cluster contained a BCCT-type transporter similar to vibrios (Fig. S5). In *Serratia*, the cluster had a toxin-antitoxin system within the cluster and was directly downstream of a type I-C CRISPR-Cas system (Fig. S5). DgcA was present in most species of the genera *Marinobacterium*, *Marinomonas,* and *Marinobacter* amongst others. The second *Vibrionaceae* branch was comprised of *Grimontia, Enterovibrio,* and *Photobacterium* clustered with DgcA from *V. penaeicida*. This was the most divergent branch on the tree (Fig. 9). These data show the catabolism region was acquired more than once within the *Vibrionaceae*. DgcA was present in all *Pseudomonas* species, and a representative number are shown in the tree, which all clustered together indicating a common ancestor (Fig. 9).

## DISCUSSION

*Vibrio natriegens* is a halophile and is non-pathogenic to humans, with a doubling time of less than 10 min under optimal growth conditions, which is proposed to be a function of its high substrate uptake rate (67, 68). Due to these attributes, there is a growing interest in using *V. natriegens* as an alternative cloning and expression tool in biotechnology (67, 71–78). Here, the osmotic stress adaptation systems were identified and characterized. We demonstrated that *V. natriegens* has high osmotic stress tolerance with an ability to grow in up to 7% NaCl without the addition of exogenous osmolytes. *Vibrio natriegens* was able to biosynthesize ectoine, glutamate, and GB as well as accumulate a variety of osmolytes utilizing BCCT transporters in response to osmotic stress. There were as many as four more BCCTs than are present in *V. parahaemolyticus,* suggesting possibly wider substrate uptake abilities and/or different substrate affinities. At 37°C, *V. natriegens* was able to survive in 7% NaCl but could not grow in the absence of NaCl. At 30°C, cells survived up to 6% NaCl and grew in the absence of NaCl. The addition of GB did not rescue growth at 37°C in the absence of NaCl. These data show that NaCl has a temperature-protective effect and that this protection is likely due to a decrease in water activity in the presence of NaCl. Choline, GB, DMG, and DMSP were shown to be highly effective osmolytes at both low and high osmolyte concentrations, rescuing growth in M9G 7.5% NaCl. GB, DMG, and DMSP were taken up efficiently by several BCCT transporters. In contrast, ectoine was only taken up by BCCT1, and sarcosine by none. In *V. natriegens*, BCCT1 took up the widest range of osmolytes, similar to what was shown for BCCT1 in *V. parahaemolyticus* (31). Previously, it was shown that BCCT1, BCCT3, and BCCT4 in *V. parahaemolyticus* were highly induced under osmotic stress conditions (31). Whereas, a recent study of *V. natriegens* showed that the ABC-type ProU (aka *choXWV*) transporters were significantly induced in 300 mM NaCl (~1.8% NaCl) compared to 0 mM, but none of the BCCT genes were induced (67).

Our data show that *V. natriegens* biosynthesized GB from choline and transported GB into the cell for osmotic stress protection at high NaCl concentrations, but unstressed cells did not. Unstressed *V. natriegens* cells grew on GB, DMG, and sarcosine as sole carbon and energy sources, but these cells could not use choline as a substrate to grow on. This suggests that choline is only directed to GB biosynthesis for osmoprotection, and the osmolarity of the growth media dictated whether GB, DMG, and sarcosine are used as nutrients or for osmotic stress protection. Interestingly, temperature also played a role in the ability to catabolize these osmolytes in *V. natriegens*. The absence of growth on GB, DMG, and sarcosine at 37°C, even in 1% NaCl, suggests that the catabolism and/or transporter genes may not be expressed at this temperature. In the rhizosphere-associated bacterium *S. meliloti*, GB is a significant osmoprotectant with uptake strongly induced under osmotic stress (43–46). GB and choline catabolism were inhibited in *S. meliloti* stressed cells, but growth on GB and choline was highly active in unstressed cells, demonstrating the importance of GB for stress protection over catabolism (43–46). This is in contrast to *P. aeruginosa*, a species that catabolizes both choline and GB in relatively high NaCl concentrations (0.75 M NaCl, which equals approx 4.25% NaCl). In *P. aeruginosa* and other Pseudomonads, it was shown that choline and GB intracellular pools are maintained under a range of growth conditions (79). In *Chomohalobacter salexigens*, only GB supported growth as a sole carbon source and only when cells were grown under high NaCl concentrations (58, 59). Our work showed that in *V. fluvialis,* DMG and sarcosine supported growth in M9 3% NaCl at 30°C and 37°C, but choline and GB did not. In addition, DMG and sarcosine had longer lag phases and lower ODs compared to *V. natriegens,* showing less efficient utilization of these substrates. We speculate that there must be significant differences in the regulation of GB, DMG, and sarcosine catabolism and/or transporter genes between these species, which will require further examination.

Analysis of the evolutionary history of the catabolism genes was revealing as it demonstrated the presence of the gene cluster with very different evolutionary histories. Within *Vibrionaceae*, the region was present in a limited number of species and was acquired more than once, reflected by its presence on different chromosomes, different gene arrangements, and different evolutionary histories. Among *Marinobacterium* and *Marinomonas* species, DgcA was prevalent with the entire cluster present in these genera, suggesting a functional GB pathway. We speculate that the acquisition of the catabolism genes provided an evolutionary advantage allowing a species a competitive advantage in specific environments such as on and within plant tissues, as animal pathogens, or associated with marine flora and fauna.

## MATERIALS AND METHODS

### Bacterial strains, media, and growth conditions

All bacterial strains and plasmids used in this study are listed in Table S2. *V. natriegens* ATCC 14048 and *V. fluvialis* 2013V-1197 were used as wild type (WT). Strains were grown in either LB (NaCl, 5 g/L, tryptone, 10 g/L, yeast extract, 5 g/L) (Thermo Fisher Scientific, Fair Lawn, NJ) final 3% NaCl (wt/vol) (LB 3% NaCl) or M9 minimal media (47.8 mM Na_2_HPO_4_, 22 mM KH_2_PO_4_, 18.7 mM NH_2_Cl, and 8.6 mM NaCl; Sigma-Aldrich) supplemented with 2 mM MgSO_4_, 0.1 mM CaCl_2_, and 20 mM D-glucose (Sigma-Aldrich) (M9G) as the sole carbon source and NaCl as indicated. Choline, GB, DMG, and sarcosine were used at a final concentration of 20 mM when used as a sole carbon source. *Escherichia coli* strains were grown in either LB supplemented with 1% NaCl (LB 1% NaCl) or M9G supplemented with 4% NaCl, as indicated. Media was supplemented with 0.3 mM diaminopimelic acid (DAP) (Sigma-Aldrich) and/or chloramphenicol (Cm) at 25 µg/mL when needed. The osmolytes GB, DMG, choline, ectoine, and DMSP were used at a final concentration of 100 µM or 1 mM. All strains were grown at either 37°C or 30°C, as indicated.

### Growth pattern analysis at varying NaCl concentrations and temperatures in M9 media in the absence and presence of compatible solutes

For growth analysis in varying salt concentrations, *V. natriegens* was grown overnight in M9G 2% NaCl at 37°C. A 2% inoculation of the overnight culture was grown to OD ~0.55 at 37°C in fresh M9G 2% NaCl. Cultures were pelleted and washed twice in M9G to remove excess salt. A 1:40 dilution into 200 µL of M9G and M9G 1% to 7.5% NaCl was made in a 96-well plate. A Tecan Sunrise microplate reader was used to incubate the plates at 37°C or 30°C with intermittent shaking and to measure the optical density at 595 nm (OD_595_) hourly for 24 h. To examine the osmolytes used by V. *natriegens*, a 1:40 dilution was inoculated into either M9G 7% NaCl or M9G 7.5% NaCl supplemented with either 100 µM or 1 mM of choline, GB, DMG, sarcosine, ectoine, DMSP, or L-proline, and the OD_595_ was measured hourly for 24 h at 37°C.

### ^1^H-nuclear magnetic resonance analysis of ectoine and GB biosynthesis

For analysis of ectoine and GB biosynthesis, *V. natriegens* ATCC 14048 was grown in M9G 1% NaCl, M9G 5% NaCl, or M9G 5% NaCl supplemented with 1 mM choline at 37°C to stationary phase. For analysis of ectoine production, no choline was added to the medium. Cultures were pelleted by centrifugation for 10 min at 6,000 rpm. The supernatant was discarded, and the pellet was washed once with an isotonic solution. Cultures supplemented with choline were washed with an isotonic solution without choline. Cultures were pelleted by centrifugation for 10 min at 6,000 rpm, and the supernatant was discarded. Cells were lysed by three freeze-thaw cycles (1 h at −80°C, then 30 min on ice). Pellets were suspended in 750 µL 190 proof ethanol and vortexed. The suspended solution was centrifuged for 10 min at 12,000 rpm to remove debris. The ethanol extract was evaporated by vacuum for 3.5 h. The extract was suspended in 700 µL deuterium oxide (D_2_O) and vortexed. Debris was removed by centrifugation for 10 min at 12,000 rpm. The extract was filter-sterilized to remove any debris not removed by centrifugation. The extract was transferred to a 5 mm NMR tube. Samples were analyzed in an AVIII 600 MHz NMR spectrometer’s qProton method with 16 scans per spectrum. Data were analyzed using MestReNova (Mnova) NMR software.

### Functional complementation of the *E. coli* MKH13 strain with *bccT* genes

Genomic DNA from *V. natriegens* ATCC 14048 RefSeq GCF_001456255.1 (chromosome I: NZ_CP009977.1 and chromosome II: NZ_CP009978.1) was used as a template to amplify *bccT1, bccT2, bccT3, bccT4, bccT6, bccT7,* and *bccT8* using the primers listed in Table S3 and designed from RefSeq NZ_CP009977.1 and NZ_CP009978.1. Primers were purchased from Integrated DNA Technologies (Coralville, IA), and each gene was PCR amplified and cloned into a pBAD 33 expression vector (80) via Gibson assembly, using NEBuilder HiFi DNA assembly master mix. Plasmids pBAVn*bccT*1, pBAVn*bccT*2, pBAVn*bccT*3, pBAVn*bccT*4, pBAVn*bccT*6, pBAVn*bccT*7, or pBAVn*bccT*8 were transformed and propagated in the *E. coli* DH5α strain (Table S2). The expression plasmids were purified and sequenced. The plasmids were then transformed into competent *E. coli* MKH13 cells (4) (Table S2). This strain contains deletions including the *betT-betIBA* genes for choline uptake and GB biosynthesis and the *putP*, *proP*, and *proU* genes required for osmolyte uptake. These deletions prevent MKH13 growth in 4% NaCl. The *E. coli* pVn*bccT*1, pVn*bccT*2, pVn*bccT*3, pVn*bccT*4, pVn*bccT*6, pVn*bccT*7, or pVn*bccT*8 strains were grown overnight in M9G 1% NaCl supplemented with 0.01% Cm to maintain the plasmid. A 1:100 dilution was made of the overnight cultures into M9G 4% NaCl supplemented with 0.01% Cm and 0.01% L-arabinose, the inducing agent, and 1 mM of GB, DMG, sarcosine, ectoine, DMSP, or L-proline. Cultures were incubated at 37°C for 24 h, and OD_595_ was measured. Statistics were calculated using the *t*-test: two-sample assuming equal variances and compared to growth with pBAD33 empty vector.

### Growth pattern analysis of *V. natriegens* and *V. fluvialis* on choline, GB, DMG, and sarcosine as sole carbon sources

*V. natriegens* ATCC 14048 and *V. fluvialis* 2013V-1197 growth on choline, GB, DMG, and sarcosine as sole carbon sources was examined at 37°C and 30°C in M9 supplemented with 3% NaCl or 1% NaCl with 20 mM of each carbon source. *V. natriegens* was grown overnight in M9 3% NaCl and 20 mM glucose at 37°C. This culture was used to generate a 2% inoculation in M9 media and grown to OD ~0.55 at 37°C and then washed twice with PBS to remove glucose. Washed cells were inoculated 1:40 into 200 µL M9 media supplemented with 3% NaCl and 1% NaCl, and 20 mM of each carbon source. Plates were incubated at 37°C and 30°C with intermittent shaking for 1 min every hour. The optical densities were measured at 595 nm every hour for 35 h for *V. natriegens* and 60 h for *V. fluvialis* using a Tecan Sunrise microplate reader and Magellan plate reader software (Tecan Systems Inc., San Jose, CA).

### Catabolism gene deletion construction

In-frame non-polar gene deletions were constructed by SOE (splicing by overlap extension) PCR and homologous recombination (81). The *V. natriegens* ATCC 14048 genome sequence RefSeq GCF_001456255.1 (Chromosome II: NZ_CP009978.1) was used to generate fragments AB and CD for each gene using the primers listed in Table S3. For *gbcA*, Gibson assembly was used to ligate the two fragments with a SacI digested suicide vector, pDS132 (82), to produce a vector with the truncated gene, pDS132VnΔ*gbcA*. pDS132VnΔ*gbcA* was transformed into the *E. coli* strain DH5α, with Cm, then into the DAP auxotroph *E. coli* strain β2155 λ*pir* (83), with Cm and DAP. This strain was used to perform conjugation with *V. natriegens*. The vector requires the *pir* gene in order to replicate. In order for pDS132∆Vn*gbcA* to replicate in *V. natriegens*, which lacks the *pir* gene, it must incorporate *pir* into the genome by homologous recombination. Colonies were grown with Cm and screened for the presence of the incorporated vector. To induce the second homologous recombination in which the truncated *gbcA* gene replaces wild-type *gbcA* in the *V. natriegens* genome, colonies were grown without Cm and in the presence of sucrose. The pDS132 vector contains the *sacB* gene, which codes for levansucrase, which will form a toxic polymer with sucrose, causing colonies that contain the plasmid to have an altered morphology with a soupy appearance. Colonies with a normal morphology were screened for the presence of the truncated *gbcA* gene. An identical protocol was followed for the construction of a *dgcA* deletion mutant. In-frame deletions were confirmed by DNA sequencing.

### Phylogenetic analysis and gene neighbor analysis

BLAST analyses were used to identify homologous protein sequences in the NCBI genome database within and outside the *Vibrionaceae* family. Proteins with >95% query coverage and >60% amino acid similarity were downloaded and aligned using CLUSTALW (84). Phylogenetic trees were constructed from 126 DgcA proteins using the program MEGA11 (85). Protein sequences were downloaded from NCBI and aligned using CLUSTALW. Trees were generated using evolutionary distances computed by the JTT matrix-based method with rate variation modelled with a gamma distribution. The pairwise deletion option was applied to all ambiguous positions for each sequence pair, resulting in a final data set comprising 698 positions.

## Data Availability

All data sets are available upon request.

## AFTER EPUB

*[This article was published on 23 April 2025 with an error in the affiliation numbering in the author byline. The error was corrected in the current version, posted on 28 April 2025.]*

## SUPPLEMENTAL MATERIAL

Supplemental material aem.00619-25-s0001.pdf Tables S1 to S3 and Figures S1 to S5.

ASM does not own the copyrights to Supplemental Material that may be linked to, or accessed through, an article. The authors have granted ASM a non-exclusive, world-wide license to publish the Supplemental Material files. Please contact the corresponding author directly for reuse.
