# Supplementary material for: Dual roles of glycine betaine, dimethylglycine, and sarcosine as osmoprotectants and nutrient sources for Vibrio natriegens
Source: Appl Environ Microbiol. 2025 Apr 23;91(5):e00619-25. doi: 10.1128/aem.00619-25 (PMC12093977; doi:10.1128/aem.00619-25)
Supplement: Supplemental material — Tables S1 to S3 and Figures S1 to S5. [file aem.00619-25-s0001.pdf]

Table S1. GB, DMG and sarcosine catabolism genes identified in *V. natriegens* with homology to *P. aeruginosa*

Annotation	Locus tag	Gene	Strand	size	%	*P. aeruginosa*
L-serine ammonia-lyase	PN96_RS22455	sdaB	-	459	68	WP_003111339.1
formyltetrahydrofolate deformylase	PN96_RS22460	purU	-	288	63	WP_003096786.1
sarcosine oxidase subunit gamma	PN96_RS22465	soxG	-	222	49	WP_003096784.1
sarcosine oxidase subunit alpha	PN96_RS22470	soxA	-	1005	67	WP_003142077.1
sarcosine oxidase subunit delta	PN96_RS22475	soxD	-	94	70	WP_003096781.1
sarcosine oxidase subunit beta family protein	PN96_RS22480	soxB	-	417	79	WP_003148382.1
serine hydroxymethyltransferase	PN96_RS22485	glyA	-	420	74	WP_003148380.1
hybrid-cluster NAD(P)-dependent oxidoreductase	PN96_RS22490	gbcB	-	371	70	WP_003096769.1
Rieske 2Fe-2S domain dioxygenase subunit alpha	PN96_RS22495	gbcA	+	399	65	WP_003096766.1
BCCT family transporter	PN96_RS22500	bccT8	-	535		
electron transfer flavoprotein subunit beta	PN96_RS22660	fixB	-	264	54	WP_003114449.1
electron transfer flavoprotein subunit alpha	PN96_RS22665	fixA	-	412	61	WP_003114448.1
Fe-S cluster-containing oxidoreductase	PN96_RS22670	dgcB	-	651	69	WP_003114447.1
NADH:flavin oxidoreductase	PN96_RS22675	dgcA	-	687	80	WP_003114446.1
DUF5943 domain	PN96_RS22680	hp	-	177	70	WP_003107282.1
dipeptidase	PN96_RS22685	pepD	-	325	80	WP_003114445.1
amidase AraC-type DNA-binding HTH domains	PN96_RS22690	glaX	-	377	59	WP_003096699.1

Table S2. Bacterial strains and plasmids used in this study

Strains or Plasmids	Genotype or Description	References
Vibrio natriegens ATCC 14048	Environmental isolate, USA	{Payne, 1961 #2348}
Δ*gbcA mutant*	ATCC 14048 Δ*gbcA*	This study
Δ*dgcA mutant*	ATCC 14048 Δ*dgcA*	This study
Vibrio fluvialis 2013V-1197	Clinical isolate, USA	CDC
Escherichia coli DH5α *λpir*	Δ*lac pir mutant*	
pBAVn*bccT* 1	pBAVn*bccT* 1 in *E. coli* DH5α *λpir*	This study
pBAVn*bccT* 2	pBAVn*bccT* 2 in *E. coli* DH5α *λpir*	This study
pBAVn*bccT* 3	pBAVn*bccT* 3 in *E. coli* DH5α *λpir*	This study
pBAVn*bccT* 4	pBAVn*bccT* 4 in *E. coli* DH5α *λpir*	This study
pBAVn*bccT* 6	pBAVn*bccT* 6 in *E. coli* DH5α *λpir*	This study
pBAVn*bccT* 7	pBAVn*bccT* 7 in *E. coli* DH5α *λpir*	This study
pBAVn*bccT* 8	pBAVn*bccT* 8 in *E. coli* DH5α *λpir*	This study
pDSΔVn*gbcA*	pDSΔVn*gbcA* in *E. coli* DH5α *λpir*	This study
E. coli MKH13	Δ*betTIBA*, Δ*putPA*,Δ*proP2*,Δ*proU Sp* ^{r}	{Haardt, 1995 #468}
MKpBAD33	pBAD33 in *E. coli* MKH13	{Gregory, 2020 #903}
MKpBAVn*bccT* 1	pBAVn*bccT* 1 in *E. coli* MKH13	This study
MKpBAVn*bccT* 2	pBAVn*bccT* 2 in *E. coli* MKH13	This study
MKpBAVn*bccT* 3	pBAVn*bccT* 3 in *E. coli* MKH13	This study
pBAVn*bccT* 4	pBAVn*bccT* 4 in *E. coli* MKH13	This study
MKpBAVn*bccT* 6	pBAVn*bccT* 6 in *E. coli* MKH13	This study
MKpBAVn*bccT* 7	pBAVn*bccT* 7 in *E. coli* MKH13	This study
MKpBAVn*bccT* 8	pBAVn*bccT* 8 in *E. coli* MKH13	This study
E. coli β2155	Δ*dapA* ::*erm pir* mutant	{Dehio, 1997 #1887}
pDS132ΔVn*gbcA*	pDS132ΔVn*gbcA* in *E. coli* β2155	This study
pDS132ΔVn*dgcA*	pDS132ΔVn*dgcA* in *E. coli* β2155	This study
pBAD33	Expression plasmid vector	{Guzman, 1995 #2353}
pBAVn*bccT* 1	RS06115 cloned into pBAD33	This study
pBAVn*bccT* 2	RS23610 cloned into pBAD33	This study
pBAVn*bccT* 3	RS04220 cloned into pBAD33	This study
pBAVn*bccT* 4	RS17645 cloned into pBAD33	This study
pBAVn*bccT* 6	RS15305 cloned into pBAD33	This study
pBAVn*bccT* 7	RS21680 cloned into pBAD33	This study
pBAVn*bccT* 8	RS22655 cloned into pBAD33	This study
pDS132	Suicide vector, R6K origin, Cm^r, sacB	{Philippe, 2004 #576}
pDSΔVn*gbcA*	pDS132 with truncated *gbcA* allele	This study
pDSΔVn*dgcA*	pDS132 with truncated *dgcA* allele	This study

Table S3. Primers used in this study

Primer Name	Sequence (5′-3′)[a]	Length[b]
VnBCCT1 (RS06115) fwd	tgggctagcgaattcgagctTGAGGAGCGTTTCAAATTAAC	
VnBCCT1 (RS06115) rev	ggatccccgggtaccgagctCTATTTGTATGCAGGTAGTTC	1660
VnBCCT2 (RS23610) fwd	tgggctagcgaattcgagctAAGGAACATTCAATGAGTAAAGATAATG	
VnBCCT2 (RS23610) rev	ggatccccgggtaccgagctTTACTTTTGGGCTCTTGGC	1690
VnBCCT3 (RS04220) fwd	tgggctagcgaattcgagctAGAGGAACGATGACAAAGG	
VnBCCT3 (RS04220) rev	ggatccccgggtaccgagctCTATTGACGCTCGGTTCTC	1621
VnBCCT4 (RS17645) fwd	tgggctagcgaattcgagctTGAGGGGAAATACACGGC	
VnBCCT4 (RS17645) rev	ggatccccgggtaccgagctTTACGTTAAACCTTGCTCTTTC	1669
VnBCCT6 (RS15305) fwd	tgggctagcgaattcgagctAAAGGACTCAATCCGACTG	
VnBCCT6 (RS15305) rev	ggatccccgggtaccgagctCTACGTTAACGCTGGTTTC	1753
VnBCCT7 (RS21680) fwd	tgggctagcgaattcgagctAGGAACCGCCTGTCAAAATAAC	
VnBCCT7 (RS21680) rev	ggatccccgggtaccgagctTTATAACCCTTGCTCTTTCGC	1738
VnBCCT8 (RS22655) fwd	tgggctagcgaattcgagctAGGAATTACTGAATACTGTTCAATATATG	
VnBCCT8 (RS22655) rev	ggatccccgggtaccgagctTTATGCTTTACGGTAAAGGTG	1699
VngbcA (RS22500) A	accgcatgcgatatcgagctTGACAAATTGGCCAGGCTTAAAG	
VngbcA (RS22500) B	attacaacttTTGTGTCATTGTCTATCTTCTTCTAG	540
VngbcA (RS22500) C	aatgacacaaAAGTTGTAATCTACAATACTTGTCTATACC	
VngbcA (RS22500) D	gtggaattcccgggagagctCTTATGCTGGCTCTCCGATG	540
VngbcA (RS22500) FL Fwd	aggttgtcgtggatccagtt	
VngbcA (RS22500) FL Rev	gctttgctcgtacgggtaaa	2560
VndgcA (RS22675) A	accgcatgcgatatcgagctTATTGAAGAAGAGATCGGTG	
VndgcA (RS22675) B	gatctttacaCGAACTGAGACATGATTTAC	540
VndgcA (RS22675) C	tctcagttcgTGTAAAGATCTGTAAGGGG	
VndgcA (RS22675) D	gtggaattcccgggagagctAACCACGTTAAACACTTC	537

[a]Lowercase letters indicate complementary regions for Gibson assembly.

[b]Length (bp) indicates the length of the PCR product for each primer pair.

A.

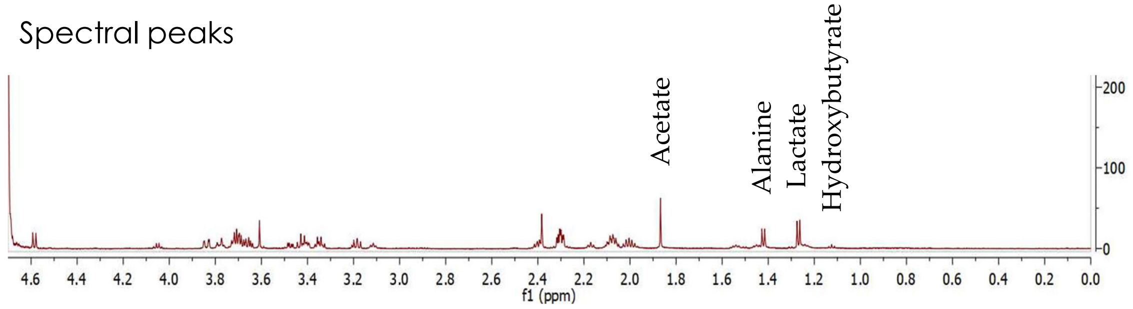

B.

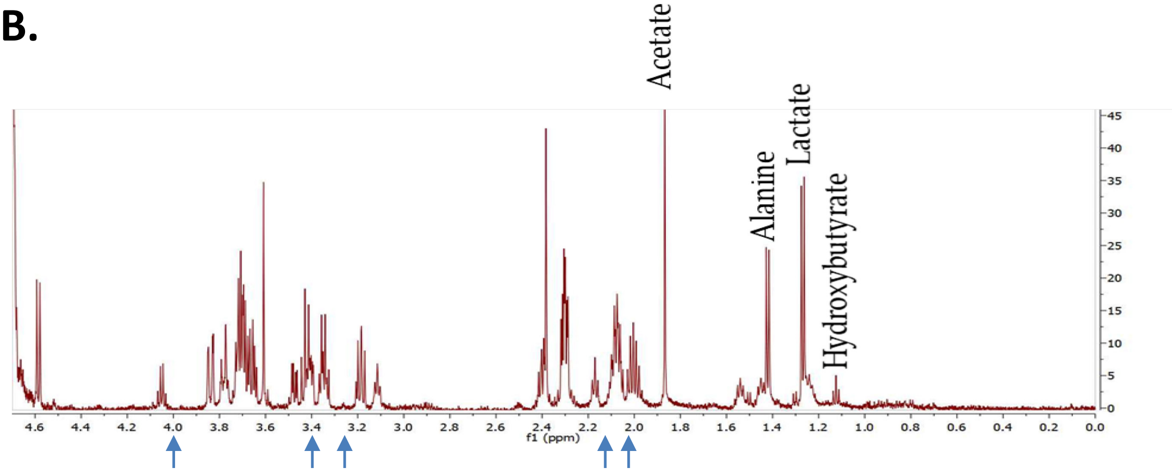

Figure S1: ^{1}H-NMR spectroscopy analysis negative control. **A.** *Vibrio natriegens* cells were grown in M9G 1% NaCl overnight and ^{1}H-NMR performed to demonstrate the absence of ectoine in unstressed cells. **B.** Scale of Y axis changed to demonstrate the absence of ectoine spectral peaks. Blue arrows indicate where ectoine spectral peaks should be.

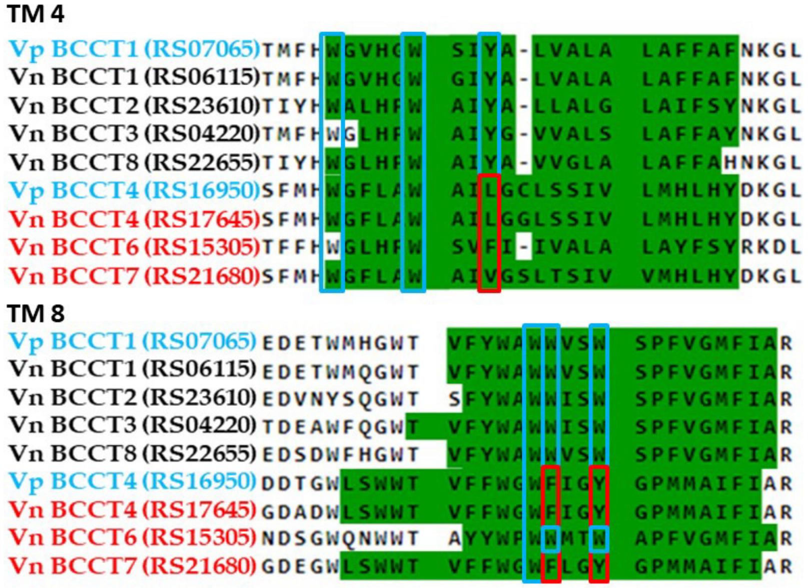

Fig. S2. **BCCT osmolyte transporter analysis**. Alignment of transmembrane (TM) region TM4 and TM8 of BCCTs substrate binding sites in BCCT1 and BCCT4 from *V. parahaemolyticus* compared to those from *V. natriegens* in the same region. Boxes indicate previously identified residues important for GB uptake. Green highlighted amino acid (AA) sequences indicate regions of importance, blue boxes indicate critical AA sites for GB uptake, red boxes indicate AA polymorphisms among the sequences examined.

A. Compatible solute biosynthesis and transporter systems in *Vibrio fluvialis*

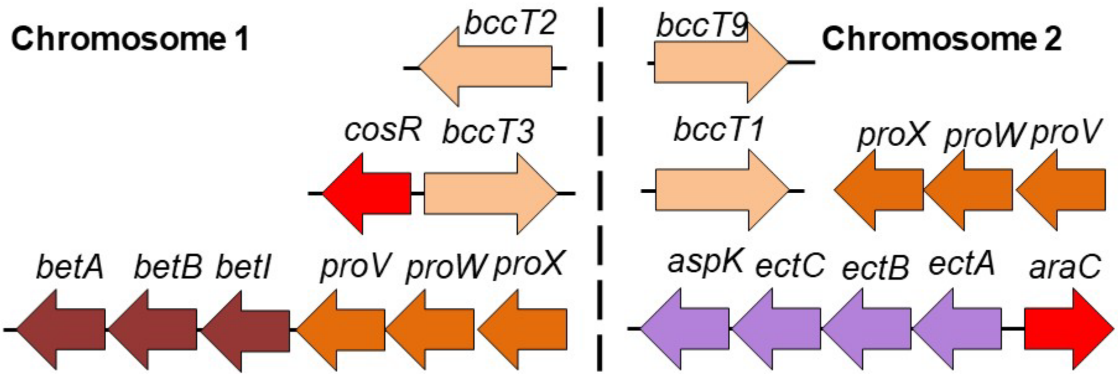

B. Osmotic stress tolerance range of *V. fluvialis*

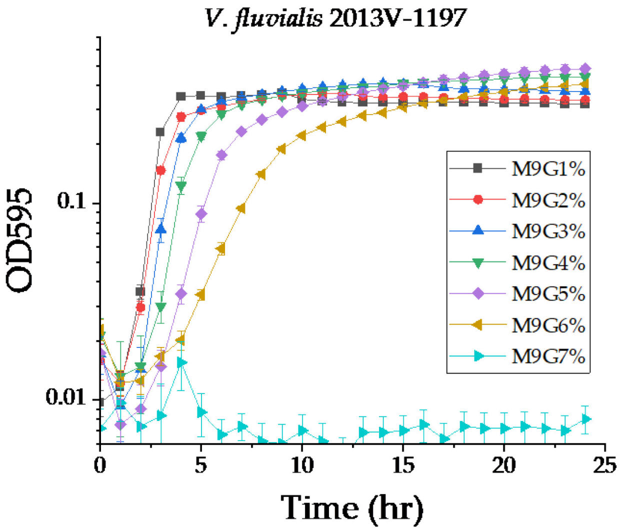

Fig. S3. **Osmotic stress response of *V. fluvialis* 2013V-1197. A**. Osmotic stress response systems present in *V. fluvialis* 2013V-1197. Arrows represent ORFs and direction of transcription. **B.** Growth curves of *V. fluvialis* 2013-1197 in minimal media (M9) supplement with glucose (M9G) with 1% to 7% NaCl at 37°C. OD was measured every hour for 24 h.

A. Growth of *V. fluvialis* in M9 3%NaCl at 30°C

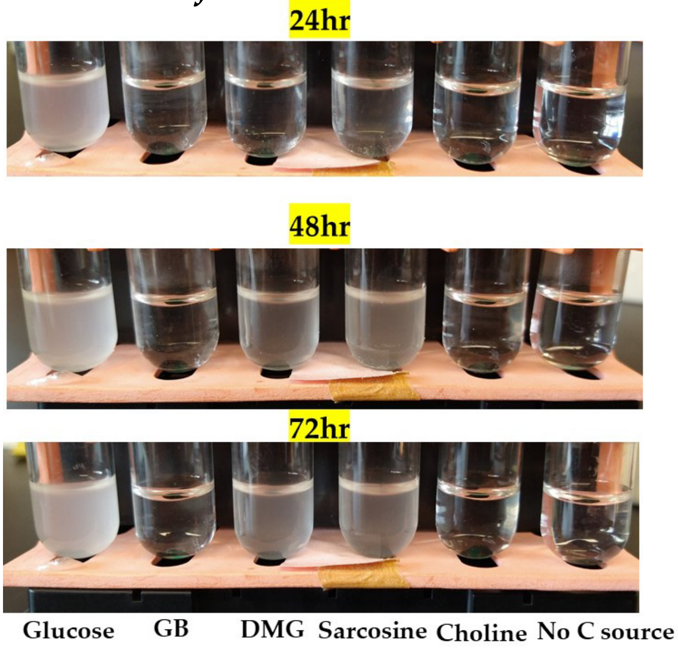

B. Growth of *V. fluvialis* in M9 3% NaCl at 37°C

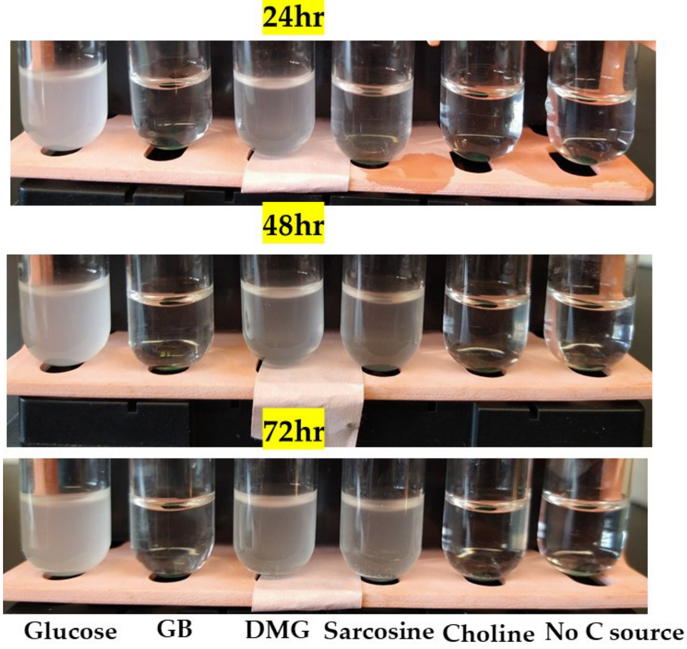

Fig. S4. Examination of *V. fluvialis* growth on GB, DMG, sarcosine, or choline as sole carbon sources in M9 3% NaCl at **A**. 30°C and **B**. 37°C. *Vibrio fluvialis* cells grown overnight in M9G 3% NaCl were washed and a 1% inoculum used to inoculate M9 3% NaCl with 20 mM of each substrate. Growth is shown in glucose, DMG, and sarcosine.

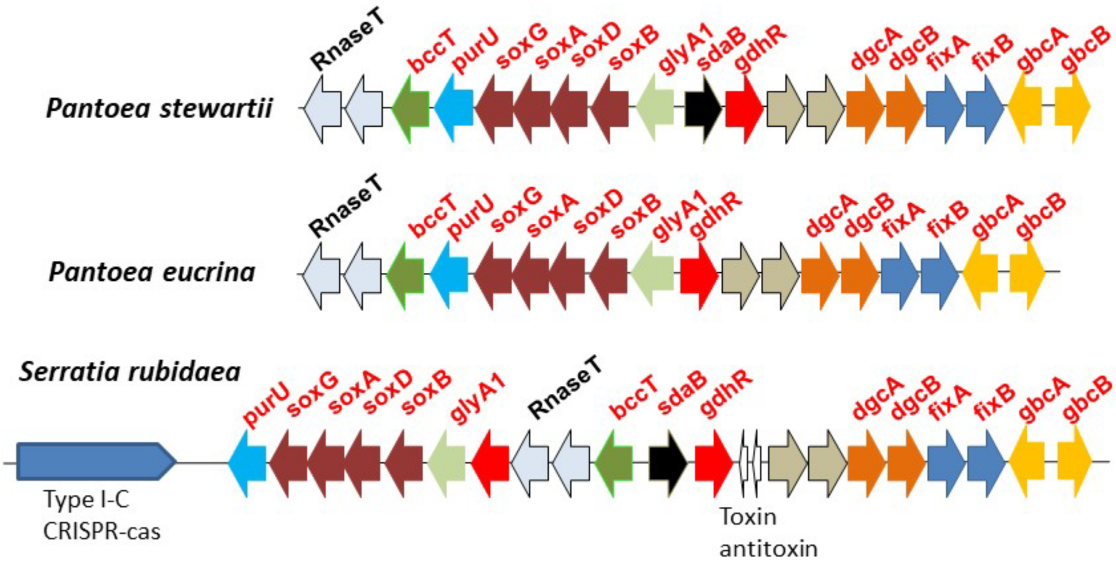

Fig. S5. Schematic of the gene order of GB, DMG, and sarcosine transporter, catabolism and regulatory genes in *Pantoea and Serratia.* Arrows indicate ORFs and direction of transcription. Similarly colored arrows indicate genes sharing a similar function,
